# A simple and rapid HPLC-UV method for the determination of valproic acid in human plasma using microwave-assisted derivatization with phenylhydrazine hydrochloride

**DOI:** 10.1016/j.heliyon.2024.e27875

**Published:** 2024-03-13

**Authors:** Sirikanya Kaewpradit, Gorawit Yusakul, Pornchai Rojsitthisak, Chutima Jantarat

**Affiliations:** a School of Pharmacy, Walailak University, Thasala, Nakhon Si Thammarat, 80160, Thailand; b Drug and Cosmetics Excellence Center, Walailak University, Thasala, Nakhon Si Thammarat, 80160, Thailand; c Functional Materials and Nanotechnology Center of Excellence, Walailak University, Thasala, Nakhon Si Thammarat, 80160, Thailand; d Department of Food and Pharmaceutical Chemistry, Faculty of Pharmaceutical Sciences, Chulalongkorn University, Bangkok, 10330, Thailand; e Center of Excellence in Natural Products for Ageing and Chronic Diseases, Chulalongkorn University, Bangkok, 10330, Thailand

**Keywords:** Human plasma, Hydrazone, Microwave assisted derivatization, Phenylhydrazine hydrochloride, Precolumn derivatization, Valproic acid

## Abstract

This study presents an efficient high-performance liquid chromatography with ultraviolet detection (HPLC-UV) method for monitoring valproic acid (VPA) level in human plasma. This method is distinguished by its simplicity, cost-effectiveness, and rapid execution, addressing the limitations associated with other advanced analytical techniques like liquid chromatography-mass spectrometry (LC-MS), gas chromatography-mass spectrometry (GC-MS), and immunoassays, which are generally complex and costly for routine application. A challenge in analyzing VPA is its non-linear protein binding profile and the absence of a chromophore in its structure, making direct detection difficult. To overcome this, the study developed an efficient HPLC-UV for VPA determination in human plasma, utilizing a simplified and rapid microwave-assisted derivatization process. Due to the lack of a chromophore in VPA structure, this work developed a microwave-assisted derivatization of VPA using phenylhydrazine hydrochloride (PH HCl). The process optimization was achieved at 450 W for 50 s, facilitating effective HPLC-UV detection. The derivatized product was characterized using ^1^H nuclear magnetic resonance (NMR) and Fourier transform infrared spectrometer (FT-IR). The derivative, identified as *(Z)-N*-phenyl-2-propylpentanehydrazonic acid, demonstrated specificity in plasma analysis with no detectable interference. The method exhibited a linear response for VPA concentrations ranging from 30 to 150 μg/mL, with a correlation coefficient exceeding 0.99. Recovery varied between 86.7% and 107%, with a maximum coefficient of variation (CV) of 10.0%. The findings suggest that the microwave-assisted derivatization technique substantially improves the feasibility and cost-effectiveness of HPLC-UV for the analysis of VPA in plasma. This method provides a viable alternative to conventional HPLC methodologies, offering a balance of efficiency and economic practicality for VPA quantification.

## Highlights

• This is the first report on quantifying valproic acid in human plasma using microwave-assisted derivatization (MAD).
• The reaction between valproic acid and phenylhydrazine hydrochloride was optimized to obtain a high-yield hydrazone product within 50 s.
• Adopting microwave-assisted thermal activation significantly reduced the reaction duration required for derivatization.
• The developed microwave-assisted derivatization (MAD) protocol offers an efficient and simple alternative for routine use in hospital and laboratory settings.

## 1 Introduction

Valproic acid (VPA), an anticonvulsant used to treat epilepsy, has a narrow therapeutic range. The recommended therapeutic range for VPA is 50–100 μg/mL [1,2]. VPA exhibits a non-linear protein binding profile [3], making it difficult to evaluate its efficacy and toxicity [4]. Therefore, VPA levels must be monitored during therapy, particularly during dosage titration. According to the United States Food and Drug Administration (US FDA) recommendations, VPA in the plasma should be measured in its parent form. Several analytical methods, including liquid chromatography-mass spectrometry (LC-MS) [5,6], gas chromatography-mass spectrometry (GC-MS) [7,8], and immunoassays [9], have been developed for the quantification of VPA. Advanced analytical methods such as LC-MS and GC-MS require highly skilled operators and are expensive for sample analysis [10]. The GC-MS method for determining VPA involved pre-column esterification, utilizing methanol as the derivatizing agent and the sample was heated at 60 °C for 30 min. This method achieved a lower limit of quantification (LLOQ) of 0.075 μg/mL, demonstrating its sensitivity and effectiveness in VPA quantification [8]. Although MS detectors offer high sensitivity for sample detection, an excessive increase in sensitivity might lead to elevated levels of interference or background noise. Immunological tests have been developed for easy and fast analysis. For example, colloidal gold-based immunochromatographic strips have been developed as a rapid qualitative detection technique for VPA [11]. Some immunoassays for quantitative purposes have also been developed, such as fluorescence polarization immunoassay (FPIA) with a sensitivity of 4.4 μg/mL [12] and enzyme-multiplied immunoassay (EMIT) with a sensitivity of 1.0 μg/mL [13]. Although these immunoassays can be used to detect low concentrations of VPA, their use in human plasma drug analysis is limited because they cannot identify the type of noise or interference [9]. Analytical methods for determining drug levels must be rapid, simple, and sensitive [14]. Therefore, high-performance liquid chromatography (HPLC) with ultra-violet (UV) spectroscopy (HPLC-UV) appears to be suitable and practical for routine drug monitoring in hospitals because of its simplicity, cost-effectiveness, and low maintenance requirements compared to LC-MS or GC-MS.

The previous study presented various HPLC-UV methods for determining VPA in biological matrices without derivatization, employing different sample preparation techniques such as liquid-liquid extraction (LLE), electromembrane extraction (EME), and solid-phase extraction (SPE). EME combined with HPLC method without derivatization provided linearity in the range of 0.5–10 μg/mL and showed a relative recovery of 75% [15]. In the LLE method, VPA was extracted using *n*-hexane under acidic conditions, followed by back-extraction with triethylamine. This approach resulted in a mean absolute recovery of 75.8% and provided linear calibration curves ranging from 1.25 to 320 μg/mL, demonstrating the validity in quantifying VPA [16]. As an example of determining VPA in other biological matrices, VPA quantification was performed using SPE for the saliva matrix, combined with an LC-UV method without derivatization. The method achieved excellent recoveries, with mean recoveries of 99.4% for VPA [17]. However, a notable limitation of SPE was that plasma contains phospholipids which strongly adhere to the reversed-phase sorbents utilized in SPE [18], leading to interference and adversely affecting the selectivity and sensitivity of the analytical method.

Owing to the absence of a chromophore in the structure of VPA and a maximum absorption wavelength (λ_max_) of 212 nm [19], its signal could interfere with solvent peaks, such as acetonitrile which has a UV cutoff of 190 nm [20]. To increase HPLC-UV method selectivity during VPA analysis, derivatization can be employed because it alters the absorption wavelength of VPA to a higher wavelength for the derivative. Liu et al. (1992) used 4-bromomethyl-7-methoxycoumarin as a derivatizing agent to derivatize VPA [21]. They found that the VPA derivative could be analyzed using HPLC-UV at 322 nm with better selectivity, sensitivity, and linearity than an enzyme immunoassay [21]. Recently, Abualhasan et al. (2020) derivatized VPA with 2-hydroxyacetophenone. The authors detected the chromophore containing VPA derivative using HPLC-UV at 254 nm [22]. The aforementioned studies were considered to be applicable to routine bioanalyses of VPA.

The conventional method used to derivatize VPA involves heating the reaction mixture for a long period. VPA was reported to be derivatized with *O*-*p*-nitrobenzyl-*N,N′*-diisopropylisourea and *p*-bromophenacyl bromide by heating the reaction at 70–80 °C for 1–1.5 h [23,24]. The use of certain derivatizing agents could reduce the temperature or heating time; for example, using 2,4′-dibromoacetophenone, VPA was successfully derivatized by heating at 70 °C for 40 min [25], while using 2-bromo-2′-acetonaphthone reduced both temperature and heating time (65 °C for 20 min) [26]. In addition, the use of catalysts has been shown to reduce reaction temperature and heating time. To this end, VPA was successfully derivatized using 2,4′-dibromoacetophenone as the derivatizing agent and tetramethylammonium hydroxide as the catalyst by heating at 55 °C for 45 min [27]. However, during HPLC analysis, column blockage may occur because of the precipitation of inorganic catalysts in the organic phase [27]. Therefore, it is preferable to avoid or minimize the use of inorganic catalysts during derivatization reactions. Although the conventional heating method can enhance the selectivity of VPA detection in plasma matrices, controlling the heating conditions and preventing contamination from the open environment is challenging. Additionally, prolonged heating at high temperatures may increase the risk of plasma matrix degradation, potentially resulting in enhanced background noise. Owing to the limitations of conventional VPA derivatization, microwave-assisted derivatization (MAD) is considered an interesting alternative.

Microwaves have been employed in the derivatization of drugs in human plasma to improve sensitivity or selectivity with a short reaction time. The reaction is performed in a closed environment, thus avoiding contamination. Tranexamic acid was derivatized with dansyl chloride under microwave conditions at 400 W for 4 min, resulting in the sensitivity of derivatized tranexamic acid being approximately 1000 times greater than that of non-derivatized tranexamic acid [28]. Phosphorus-containing amino acid herbicides were derivatized with trimethylorthoacetate under microwave conditions of 700 W for 5 min, which significantly reduced the reaction time from 30 to 5 min [29]. Previous evidence has suggested that MAD is appropriate for addressing the drawbacks of conventional methods as it presents an interesting alternative heating technique for derivatization. MAD has been explored for the derivatization of VPA for its analysis in pharmaceutical formulations [30]; however, to date, no studies have reported the use of MAD for the analysis of VPA in human plasma. Therefore, the objective of this study was to examine the feasibility of analyzing VPA in human plasma by enhancing its selectivity through derivatization using MAD to shorten the reaction time during sample preparation, to ultimately support routine therapeutic monitoring of VPA in hospitals. To enhance the selectivity of VPA by reducing plasma matrix or solvent peak interference, phenylhydrazine hydrochloride (PH HCl) was chosen as the derivatization agent in this study. PH HCl has been successfully used to derivatize octanoic acid, which has the same number of carbon atoms as VPA, through MAD in non-plasma matrices. The condition was set at 400 W for 1 min. The results demonstrated a seven-fold improvement in sensitivity and selectivity of the derivatized samples compared to the signal obtained from the non-derivatized samples [31].

The feasibility of the PH HCl and VPA derivatization reaction using a microwave oven as the heating method under moderate conditions was examined for the first time in this study. The microwave conditions were optimized to obtain the highest ratio of the derivatized product to the remaining reactant. Next, the linearity between the amount of derivatized product formed and the added VPA was assessed to ensure the reaction proceeded in the desired direction. The derivatized product was synthesized and purified through SPE to elucidate its chemical structure using ^1^H nuclear magnetic resonance (NMR) and Fourier transform infrared spectrometry (FT-IR), and was used as a standard for determining the specificity of the reaction in human plasma. Finally, the bioanalytical method using an HPLC-UV detector to measure VPA in human plasma via the MAD method was developed and validated, considering various parameters, including specificity, the lower limit of quantification (LLOQ), calibration curve and linearity, accuracy, and precision.

## 2 Materials and methods

### 2.1 Chemicals, materials and reagents

Sodium valproate (SVP) with a purity of 98% and VPA secondary standard certified reference material (99.5%) were purchased from Sigma-Aldrich (Saint Louis, MO, USA). PH HCl as a derivatizing agent was obtained from Loba Chemie (Maharashtra, India). Fresh frozen plasma (FFP) was acquired from the Thai Red Cross Health Station (Thung Song, Nakhon Si Thammarat, Thailand). This study was approved by the Ethics Committee in Human Research at Walailak University (Ethical Approval Number: WUEC-23-194-01). Acetonitrile and methanol (both HPLC grade), used as solvents for HPLC analysis, were purchased from RCI Labscan Limited (Bangkok, Thailand). All other chemicals and reagents used were of analytical grade. All molecular structures used in the studies were drawn in ChemDraw 22.2.0. The publication schematic diagrams were created from BioRender software (BioRender.com).

### 2.2 Chromatographic conditions

To monitor the derivatization reaction and measure VPA in the plasma following derivatization, the established optimum chromatographic conditions were as follows: The mobile phase consisted of acetonitrile-monobasic sodium phosphate in deionized water (pH 3.14; 0.03 M) at a ratio of 40:60, v/v. Considering the pKa of SVP (5.14), phosphoric acid was used to adjust the pH of the aqueous medium to 3.14 to prevent peak tailing. Chromatographic analysis was performed using an Ultimate 3000 HPLC system (Thermo Scientific, Waltham, Massachusetts, USA). The injection volume was 10 μL with a flow rate of 0.5 mL/min. Separation was performed on a Hypersil BDS C18 column (4.6 × 250 mm, 5 μm) (Thermo Fisher Scientific Inc, Massachusetts, USA). The chromatograms were recorded at 277 nm. These chromatographic conditions were employed to monitor the derivatization reaction and measure valproic acid in plasma following derivatization.

### 2.3 Derivatization reaction and optimization of microwave conditions

Sodium valproate (SVP, 0.5 mg/mL) was mixed with PH HCl (2.51 mg/mL) in a reaction medium consisting of acetonitrile and deionized water at a ratio of 90:10 in a 25 mL volumetric flask, after which the mixture was brought to volume. Acetonitrile, commonly used for precipitating protein from plasma before HPLC analysis [28], was chosen as the reaction medium to examine the feasibility of the reaction in its presence. Three milliliters of the mixed solution was then transferred to a 10 mL beaker. The derivatization reaction was carried out by heating in a microwave oven (MS28J5255UB, Samsung, Chonburi, Thailand).

To optimize the microwave conditions, the irradiation time (40, 50, and 60 s) and power (300, 450, and 600 W) were varied. The heating step was conducted intermittently, alternating between 5 s of heating and 10 s of no heating to prevent overheating. Subsequently, the sample solution (200 μL) was diluted with the HPLC mobile phase, filtered, and analyzed using the HPLC system. The optimum microwave conditions were selected based on the maximum area under the curve (AUC) ratio of the derivatized product to the remaining PH HCl.

To confirm that the optimal microwave conditions were effective across a range of SVP concentrations, various concentrations of SVP (from 0.5 to 2 mg/mL) were tested using a constant amount of PH HCl (2.51 mg/mL) and reaction medium (acetonitrile and deionized water in a 90:10 ratio). The correlation between SVP concentrations and the ratio of the derivatized product to the remaining PH HCl peak area was assessed.

### 2.4 Synthesis and purification of derivatized product

This experiment was conducted to obtain a purified derivatized product, a hydrazone compound, for use as a standard for bioanalytical method validation. After the reaction was completed, the derivatized solution was evaporated using a rotary evaporator (IKA RV10 Digital V, Bec Thai Bangkok Equipment & Chemical Co., Ltd, Nakhon Pathom, Thailand) at a rotation speed of 130 rpm, a bath temperature of 40 °C, and a pressure of 165 mbar to remove acetonitrile from the reaction solution.

To purify the derivatized product from the reaction solution, SPE was performed. The derivatized solution was dissolved in the SPE mobile phase to facilitate its transfer from the round bottomed flask. Considering a column diameter of 10–13 mm, the appropriate volume of the eluent was determined to be 100 mL. Cosmosil C18-OPN packing material (Nacalai Tesque, Inc., Kyoto, Japan) was conditioned with methanol overnight before being loaded into the SPE-column, which utilized a 20 mL measuring pipette (HBG™, Mainz, Germany). This was followed by rinsing with 100 mL methanol and deionized water. Subsequently, the eluent and sample solution from the derivatization step were subjected to column chromatography. The eluent ratio was then optimized. To collect fractions, 20 mL of the solution was intermittently collected in each fraction. These fractions were then diluted and injected into the HPLC system to determine which provided the purest derivatized product. The selected fraction had the remaining organic solvent removed using a rotary evaporator, and any residual deionized water was removed using a freeze-dryer (Christ, GAMMA 2–16 LSC, Osterode am Harz, Germany). The percentage purity of the derivatized product was calculated using Equation (1).

(1) $$\%\;purity=\left(S_{dev}/S_{total}\right)\times 100$$

Where *S*_*dev*_ is area under the curve of derivatized product and *S*_*total*_ is the total area.

The yield of the derivatized product was calculated according to Equation (2) provided below:

(2) $$\text{Yield}\left(\%\right)=\left(A_{p\;}/T_{p}\right)\times 100$$

Where $A_{p}$ is actual amount of product and $T_{p}$ is theoretical amount of product.

### 2.5 Elucidation of derivatized product

#### 2.5.1 Nuclear magnetic resonance spectroscopy (NMR)

To elucidate the structure of the derivatized product, the ^1^H NMR *(*Bruker, Ascend500, Avance NEO, Germany*)* was used. The derivatized product (1 mg) was dissolved in deuterium oxide. NMR parameters including the number of protons, chemical shift, integral, and splitting pattern were used to predict the molecular structure.

#### 2.5.2 Fourier transform infrared (FT-IR) spectroscopy

FT-IR spectroscopy with attenuated total reflectance (ATR) (Tensor27, BRUKER, Germany) was used to analyze the functional groups present in the reactant and derivatized product. The analysis involved 16 scans across the wave number range of 3396.37–399.251 cm^−1^ with a resolution of 4. Subsequently, the spectra of SVP, PH HCl and the derivatized product were determined.

### 2.6 Plasma sample preparation

To determine whether the developed HPLC-UV method (in Section 2.2) could effectively analyze VPA in human plasma after derivatization with PH HCl using the optimized MAD conditions, plasma samples were prepared, and protein precipitation was used as part of the sample preparation. Acetaminophen (C_8_H_9_NO_2_), which contains a chromophore and provides an appropriate retention time for the derivatized product, was used as the internal standard. It was dissolved in deionized water to a concentration of 110 μg/mL. Subsequently, 100 μL of the internal standard solution was spiked into each 500 μL plasma sample. Acetonitrile (500 μL) was added to the plasma to precipitate the proteins. The mixture was then vortexed at 3200 rpm for 1 min and centrifuged at 2896 g for 15 min (Refrigerator Centrifuge Eppendorf, 5810R). The supernatant was then transferred to another centrifuge tube, and PH HCl (30 μg/mL) was added. An additional 4500 μL of acetonitrile was added to achieve the ratio of reaction medium (acetonitrile to deionized water; 90:10, respectively) in a 10 mL beaker. The sample solution was then heated in a microwave oven at 450 W for 50 s in intermittent steps to prevent overheating, alternating between 5 s of heating and 10 s of no heating. After heating, 300 μL of the sample solution was diluted with deionized water to 1000 μL, filtered, and injected into the HPLC system. The HPLC conditions used to analyze the derivatized VPA product in plasma were the same as those described in Section 2.2.

### 2.7 HPLC-UV method validation

#### 2.7.1 Specificity

To ensure that the analysis of the derivatized product resulting from the derivatization reaction in human plasma did not interfere with any other substances, a specificity test was performed. The chromatograms of blank human plasma and zero plasma blank (blank plasma spiked with internal standard) were compared with the chromatogram of the sample solution. The sample solution was prepared by adding 500 μL of human plasma into a 1.5 mL centrifuge tube. Standard VPA at a concentration of 30 μg/mL (LLOQ) was added. The sample solution was added with the internal standard solution, and the samples were prepared as described above.

#### 2.7.2 LLOQ and calibration curve

According to the bioanalytical method validation guidance for industry, blank plasma (plasma without analyte and IS), zero plasma blank (blank with IS) and at least six sample concentrations (plasma containing VPA and IS) were prepared to construct the calibration curve. In this study, the calibration curve was prepared to cover the therapeutic range of VPA in plasma (50–150 μg/mL) [32]. The LLOQ was established to be less than 50 μg/mL; thus, the concentrations used in this experiment were 30, 45, 60, 75, 120, and 150 μg/mL by adding different volumes of VPA standard solution to the plasma to create these concentrations. Five hundred microliters of the prepared calibration sample were added with a constant volume of internal standard and then the sample preparation was carried out as in plasma sample preparation. The calibration curve was constructed on three separate days to investigate the precision and accuracy at each concentration level. The AUC of the derivatized product in the plasma relative to the internal standard (y-axis) and VPA standard concentration (x-axis) were plotted to produce the calibration curve. The LLOQ was defined as the lowest concentration on the calibration curve that could be measured with acceptable precision and accuracy [9]. The acceptance criteria for accuracy were 80–120% for the LLOQ and 85–115% for other nominal concentrations. The acceptance criteria for precision were that the coefficient of variation (%CV) should not exceed 15% for the nominal concentration and 20% for LLOQ.

#### 2.7.3 Accuracy and precision

Since VPA has been reported to exhibit acceptable therapeutic effects at serum concentrations of 50–150 μg/mL [32,33], the accuracy and precision were performed at four concentration levels. These included the LLOQ (30 μg/mL), low quality control (LQC) at 65 μg/mL, medium quality control (MQC) at 75 μg/mL, and high quality control (HQC) at 120 μg/mL. Five replicates were prepared for each concentration. Subsequently, the accuracy at each concentration was determined (should be within ±15% for the nominal concentrations, except for the LLOQ, which should be within ±20%). Additionally, precision at each point should not exceed 15% CV except for the LLOQ (±20% CV).

### 2.8 Greenness assessment and Blue Applicability Grade Index (BAGI) evaluation

Green Analytical Procedure Index (GAPI) was used to determine the impact of analytical methods on the environment and workers [34] and Blue Applicability Grade Index (BAGI) was used to determine the practicality of analytical procedures [35]. Both of the free available software were downloaded from https://mostwiedzy.pl/complexgapi for GAPI [36] and https://mostwiedzy.pl/bagi for BAGI [35].

## 3 Results and discussion

### 3.1 Chromatographic conditions

**Fig. 1:**
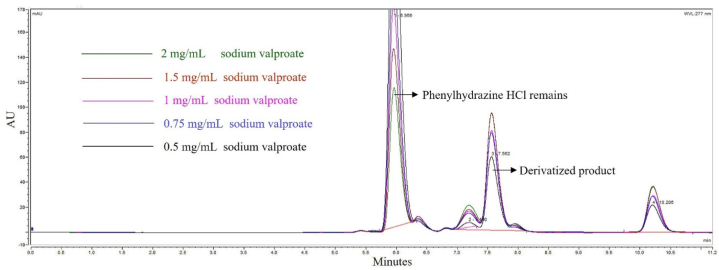
HPLC chromatograms of the derivatized products resulted from various concentrations of SVP.

The reaction between PH HCl and SVP in acetonitrile, as the reaction solvent, when assisted with microwave, can yield the derivatized product as shown in Fig. 1. The peak of the derivatized product, (*Z*)-*N*-phenyl-2-propylpentanehydrazonic acid, was observed at a retention time of 7.6 min, while PH HCl eluted at approximately 5.9 min. This method provided optimal selectivity due to a resolution at the peak of the derivatized product was 8.12, which is greater than 1.5. Additionally, the number of theoretical plates was 15,748 and the asymmetry of the derivative peak was 1.27. All the parameters met the acceptance criteria, which are the resolution greater than 1.5, a number of theoretical plates greater than 2000, and asymmetry peak below 2 [37]. This indicates that the derivatized product could be formed in a medium containing acetonitrile, which is an important solvent used to deproteination VPA from human plasma [38]. As the reactant did not completely dissolve in pure acetonitrile, sufficient water was required to facilitate the reaction. The optimum medium for product formation was determined to be acetonitrile and deionized water in various ratios, with 90:10 being suitable. To quantify the derivatized product and the remaining PH HCl, the UV detector was set at 277 nm, which was found to be appropriate.

### 3.2 Derivatization reaction and optimization of microwave conditions

**Fig. 2:**
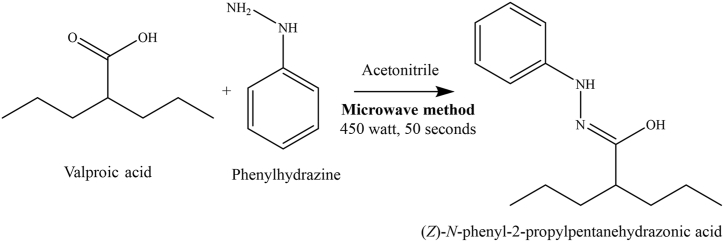
The proposed reaction mechanism between sodium valproate (SVP) and phenylhydrazine hydrochloride (PH HCl) leading to the formation of a hydrazone compound.

SVP, the active ingredient in drug formulation, is available in various dosage forms, including tablets, enteric-coated tablets, slow-release forms, parenteral solutions, and oral solutions. In the acidic environment of the stomach, SVP is converted to VPA. Protons originating from the hydrochloride of phenylhydrazine convert SVP into VPA. The reaction between PH HCl and VPA involves hydrazone formation, which is the reaction between hydrazine and the carbonyl group, as shown in Fig. 2.

**Fig. 3:**
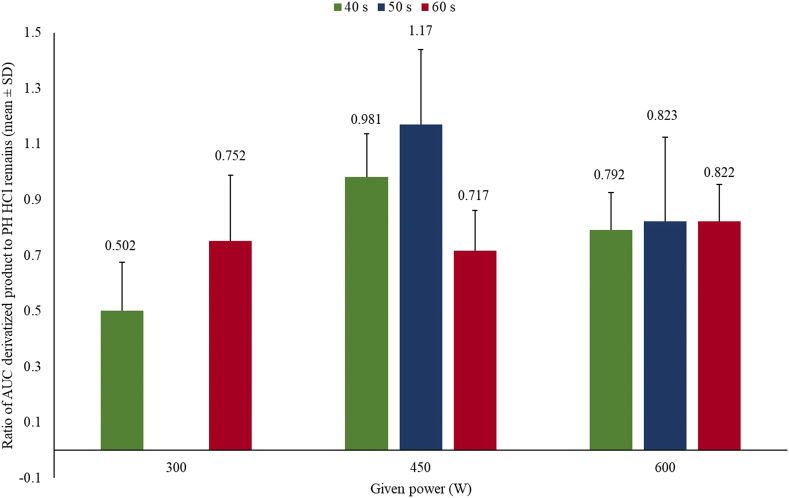
Optimization of the condition of microwave-assisted method (MAD) (*n* = 3).

To determine the optimal microwave conditions, including the power and irradiation time, these parameters were varied. As shown in Fig. 3, the conditions that provided the most derivatized product were 450 W for an irradiation time of 50 s. The reaction time for the derivatized product using MAD in this work was shorter than that of previous reported conventional heating methods [[23], [24], [25], [26], [27]]. The difference is that the conventional methods heat from the core of the sample through conduction or convection. In contrast, the microwave method irradiates the entire volume of the sample homogenously, or causes localized heating at the centers of polar molecules, resulting in rapid heating [39].

The optimum microwave condition of 450 W for 50 s was effective to facilitate the reaction between the derivatized product relative to PH HCl remains and the amount of standard VPA (0.5–2 mg/mL). A linear relationship was achieved and was presented in the equation y = 0.4457x + 0.0903, where y is the ratio of the AUC of the derivatized product to the remaining PH HCl and x is the concentration of SVP with a correlation coefficient (*r*) of 0.9905, indicating a significant linear relationship between the two variables. As the concentration of SVP (x-axis) increases, the ratio of the AUC of the derivatized product to the remaining PH HCl (y-axis) also increases. This equation provided the residual plot with a random pattern scattered around zero, meaning the model fits the data well. The *p*-value of the y-intercept is 0.1394, which is greater than 0.05, indicating that the y-intercept is not statistically different from zero.

Additionally, one important point of this study was to determine the minimum concentration of PH HCl while the reaction with VPA to proceed. According to the law of definite proportions based on Equation (3), the ratio of reactant was sufficient at 1:1.

(3) $${\;C}_{8}H_{15}\text{Na}O_{2}+C_{6}H_{8}N_{2}\;\cdot \;\text{HCl}\;\to \;{\;C}_{14}H_{22}N_{2}O+\text{NaCl}+H_{2}O$$

Hence, this study used PH HCl at a concentration of 17.34 mmol/L derivatized with an SVP concentration of 12.03 mmol/L. This ratio of the reactant was found to be sufficient for facilitating the reaction and minimizing the residual PH HCl, thus reducing chemical waste. B. Bravo et al. (2004) derivatized phenylhydrazine (PH) with octanoic acid, which has the same number of carbon atoms as VPA, resulting in a seven-fold enhancement in sensitivity and selectivity. The concentration of PH was 1.99 mmol/L, while the concentration of octanoic acid was 0.0591 mmol/L [31]. The greatest yields were achieved when the derivatizing agent was used in excess [40].

### 3.3 Synthesis and purification of derivatized product

The SPE method, employed as a purification technique, was found to be effective in removing impurities and contaminants from the derivatized product. The purity of the derivatized product, as determined by HPLC, was 96.41%. The optimal eluent was found to be 5% acetonitrile in deionized water. Therefore, this purified derivatized product was chosen to be used as a standard for spiking into human plasma during the specificity test. Regarding the percent yield of the derivatized product as a pure substance, the theoretical yield was initially established at 12.04 mmol/L. After undergoing the synthesis and purification process, the actual yield of the product was found to be 0.8414 mmol/L. Consequently, the calculated percentage yield of the derivatized product was approximately 6.99%. The observed lower yield for the derivatized product was primarily attributed to the stringent purification process employed, specifically solid-phase extraction (SPE). Therefore, the process involved removing the fractions that contain the high-impurity substance resulting in significantly reducing the overall yield.

### 3.4 Elucidation of derivatized product

**Fig. 4:**
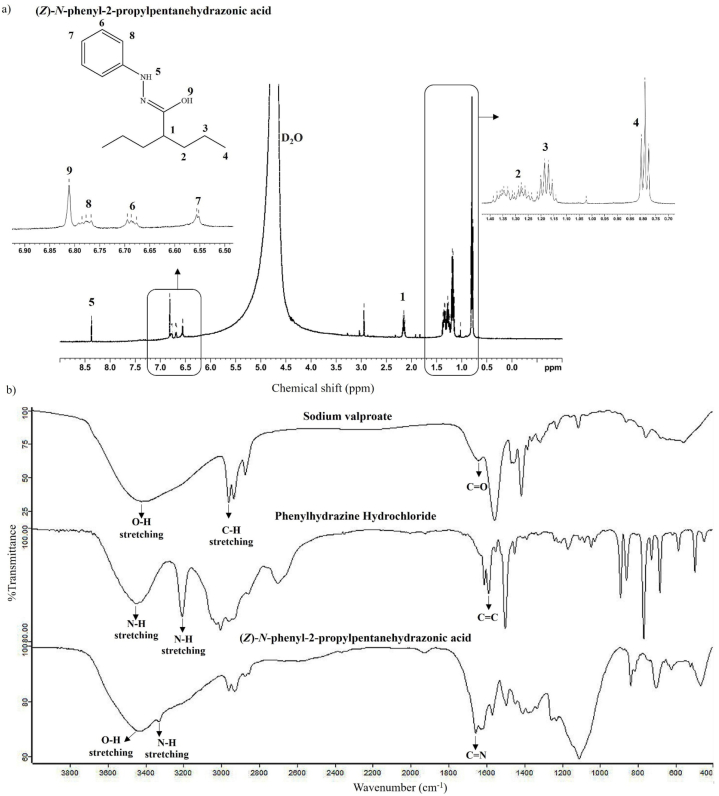
Elucidation results including a) NMR spectrum of (*Z*)-*N*-phenyl-2-propylpentanehydrazonic acid or the derivatized product and b) FTIR spectra of the reactant and derivatized product.

From the elucidation results, the derivatized product was identified as (*Z*)-*N*-phenyl-2-propylpentanehydrazonic acid. The occurrence of hydrazone (C=N) and hydrazine (H_2_N–NH_2_) bonds ensured the formation of the derivatized product. All ^1^H signals were interpreted in terms of their chemical shifts (δ), splitting patterns, and signal intensities. Fig. 4a depicts the NMR spectrum of the synthesized product. There were nine signals, indicating that seven protons were connected to carbon atoms, one proton was connected to the hydrazine bond, and one proton was connected to the hydroxyl group. The chemical shift of the hydrazide structure (N–H) [41] occurred at δ8.37 (*s*, NH-5). The hydroxyl group occurred at δ6.81 (*s*, OH-9). The signals of the aromatic system occurring at the downfield area were δ6.55 (*d*, *J* = 5 Hz, Ar-7), δ6.69 (*dd*, *J* = 5 Hz, Ar-6), and δ6.78 (*dd*, *J* = 5 Hz, Ar-8). The signals of the alkane structure occurred at δ2.15 (*m*, CH-1), δ1.17(*m*, CH_2_-3), δ1.30(m, CH_2_-2), and δ0.79 (t, CH_3_-4). The solvent peak of deuterium dioxide appeared at δ4.71 [42].

To support the formation of (*Z*)-*N*-phenyl-2-propylpentanehydrazonic acid, the IR spectrum in Fig. 4b shows that the reaction between the carboxylic functional group and hydrazine yields a hydrazone compound. The C=N stretching occurs in the region around 1660 cm^−1^, indicating a covalent bond formation between VPA and PH HCl. The carbonyl group (C=O) found at 1640 cm^−1^ in VPA was absent. The amine group (N–H) of derivatized product was presented at 3329 cm^−1^.

### 3.5 HPLC-UV method validation

#### 3.5.1 Specificity

**Fig. 5:**
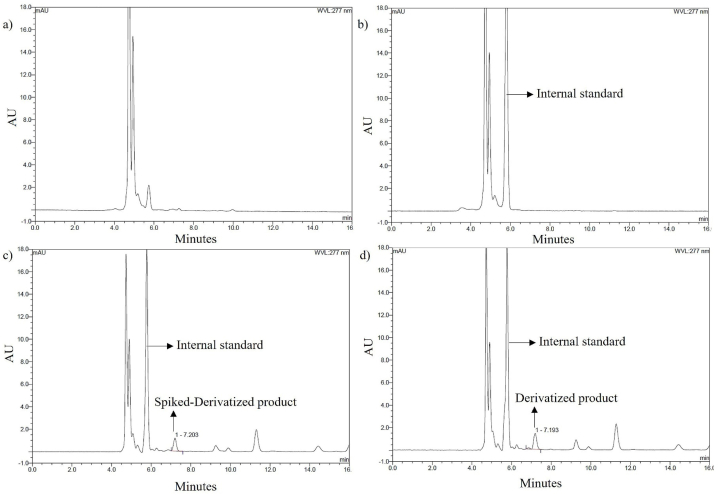
Chromatograms of (a) blank plasma, (b) zero blank plasma, (c) plasma with standard addition of the derivatized product, and (d) plasma following the derivatization of the standard VPA concentration of 30 μg/mL.

The specificity of the HPLC-UV method for analyzing the VPA derivative in plasma was measured, with results shown in Fig. 5d. The peak of the derivatized product resulting from the derivatization reaction in plasma did not interfere with any other substance, such as the plasma matrix. Furthermore, the retention time of the product from the derivatization reaction in plasma corresponded to the retention time of the product from standard addition (Fig. 5c). This supports that the product formed in the plasma was (*Z*)-*N*-phenyl-2-propylpentanehydrazonic acid. Compared with the chromatogram of non-derivatized VPA from previous studies [16,38,[43], [44], [45]], hydrazone formation enhanced the selectivity of the derivatized product from plasma.

#### 3.5.2 LLOQ and calibration curve

Three sets of calibration curves were constructed to examine the range and correlation coefficient (*r*) on individual days. As seen in Table 1, the calibration curves were linear between 30 and 150 μg/mL, and the correlation coefficient obtained from the three calibration curves was greater than 0.99. This indicates that the concentration of standard VPA (x-axis) and the ratio of the product formed in the plasma to the internal standard (y-axis) were directly proportional. The LLOQ, the lowest concentration on the calibration curve that provides satisfactory precision and accuracy with acceptance criteria [9], of this analytical method was established to be 30 μg/mL. Given that the therapeutic range of VPA in human plasma is 50–100 μg/mL [1,2], the sensitivity obtained from this analytical method was adequate for determining VPA in plasma. However, the increased sensitivity may lead to increased interference or background noise. Additionally, the accuracy at each concentration was within 85–115% of the nominal concentrations, and the accuracy at the LLOQ was within 80–120%, meeting the acceptance criteria outlined in the bioanalytical method validation guidance for industry.

**Table 1:** Linearity results obtained from three independent runs over consecutive days.

Days	Calibration equation	Correlation coefficient *(r)*
1	y = 0.0025x – 0.0542	0.9977
2	y = 0.0003x – 0.0077	0.9960
3	y = 0.0010x – 0.0163	0.9941

#### 3.5.3 Accuracy and precision

The accuracy obtained from standard VPA concentrations of 65, 75, and 120 μg/mL was within the ranges of 86.3–92.7%, 85.1–106%, and 85.4–89.6%, respectively (Table 2). These findings met the FDA criteria [46], as they were within the range of 85–115%. The precision results (%CV) obtained from each concentration were also within the accepted criteria (%CV ± 15). Furthermore, the accuracy of the LLOQ (30 μg/mL) was between 101 and 111%, which was within the range of 80–120%, and the precision results met the acceptance criteria (%CV ±20) as stipulated in the FDA guidelines [46].

**Table 2:** Accuracy and precision of valproic acid (VPA) determination VPA in human plasma.

Sample^a^ (*n* = 5)	Concentration added (μg/mL)	Concentration Found (μg/mL), mean ± SD	Mean recovery (%)	Precision (%CV)
LLOQ	30	32.15 ± 1.13	107	3.50
LQC	65	58.15 ± 1.54	89.5	2.64
MQC	75	68.77 ± 6.90	91.7	10.0
HQC	120	104.0 ± 2.24	86.7	2.15

a LLOQ**:** lower limit of quantification; LQC**:** low quality control; MQC**:** medium quality control; HQC**:** high quality control.

### 3.6 Comparison of MAD with other methods for determining VPA in human plasma

The method for determining VPA in human plasma using the MAD technique in this work can reduce the reaction time compared to other methods as summarized in Table 3. It provides adequate linearity (30–150 μg/mL) for the quantification of VPA in human plasma, aligning with the therapeutic range of VPA in human plasma (50–100 μg/mL) [1,2]. Additionally, this method improves selectivity as the resolution at the peak of the derivatized product was 8.12, which is greater than 1.5 [37], and avoids interference from the plasma peak compared to the non-derivatization methods [38].

**Table 3:** Comparison of MAD with other methods for determining VPA in human plasma.

Derivatizing agent	Reaction time	Heating condition^a^	Linear^b^ (μg/mL)	Recovery (%)	references
**Non-derivatization**	–	–	10–150	94.3^c^	[38]
**Conventional method derivatization**					
4-brophenacyl bromide	15 min	70 °C,	274–372^d^	95–100	[47]
*O-p*-Nitrobenzyl-*N, N′* diisopropylisourea	1.5 h	80 °C,	50–100	97.7^c^	[23]
*para*-bromophenacyl bromide	1 h	70 °C	10–150	25.02 ± 1.5^c^	[24]
2,4′-dibromoacetophenone	45 min	55 °C	1.0–200.7	91.6–97.4	[27]
**Microwave-assisted derivatization (MAD)**					
Phenylhydrazine hydrochloride	50 s	450 W	30–150	86.7–107	This work

a The water bath's heating was indicated by temperature, whereas the microwave's heating was indicated by power.

b The valproic acid concentration in the plasma/serum.

c The value was calculated from average percent recovery.

d The unit was μmol/L.

### 3.7 Greenness assessment and Blue Applicability Grade Index (BAGI) evaluation

The concept of Green Analytical Chemistry (GAC) focuses on minimizing the use of toxic solvents in sample preparation and extraction steps, as well as to reduce the energy consumption of analytical instrument [34]. This study aims to determine the ecological impact of analytical procedures by using GAPI.

**Fig. 6:**
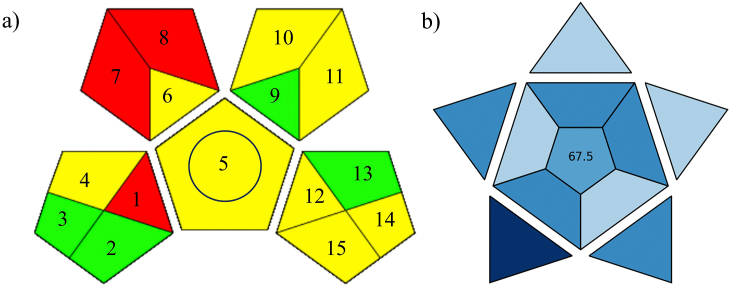
The tools for the determination of the analytical method, including a) the GAPI pictogram, and b) the BAGI index pictogram.

GAPI, the software that assesses the green character of analytical methods across various aspects, including sample preparation, reagents and solvents used, waste production, sample collection, and instrumental consumption [36,48]. This tool presents results in the form of a five-pointed star diagram (pentagram). The three colors including green, yellow, and red indicate the method's environmental impact severity [36]. In the GAPI pictogram analysis shown in Fig. 6a, the significant presence of the yellow zone relates to the use of acetonitrile (area 5, 10, and 11), while the red zone relates to the derivatization process in sample preparation (area 7 and 8). However, this study performed the derivatization reaction using MAD, which avoids the drawbacks of traditional methods. Additionally, the study employed HPLC with a low flow rate (0.5 mL/min), significantly reducing energy consumption by the LC system. Consequently, area 12 is marked in yellow.

BAGI is software that assesses the practicality of analytical procedures. It evaluates various metrics, including the type of analysis, sample preparation techniques, number of steps involved, samples analyzed per hour, sample volume, required preconcentration, instruments needed, and the level of automation [35]. As indicated in Fig. 6b, the total score derived from the BAGI software for the analytical method in this study was 67.5. This score falls within the established acceptance criteria [35], which typically range from 25 to 100. Scores below 25 indicate that the method is less applicable, while a score of 100 represents excellent applicability [35]. This score was calculated based on various factors, including the type of analytical method used in this study, which is a quantitative analysis of VPA in human plasma using MAD. The analytical method allowed for the simultaneous preparation of multiple samples through protein precipitation and derivatization. Each sample contained approximately 500 μL of plasma, ensuring adequate sensitivity and minimal biological waste. The method utilized common commercial solvents such as acetonitrile and reagents not commonly used in laboratories but prevalent in commercial settings such as PH HCl. The analysis was conducted using HPLC, a standard instrument in the laboratory. The HPLC autosampler played a crucial role in reducing human error and minimizing the risk of contact with biological fluid samples.

## 4 Conclusions

In conclusion, this study has successfully pioneered a practical and efficient MAD HPLC-UV method for the quantification of VPA in human plasma, in line with FDA validation standards. Demonstrating robust specificity, linearity, and precision, the MAD approach significantly simplifies the sample preparation process by omitting traditional nitrogen gas drying, thus streamlining the protein precipitation stage. The optimal MAD conditions, set at 450 W for 50 s, facilitated rapid and reproducible derivatization, ideal for routine clinical application and well-suited for hydrazone formation. Due to the therapeutic range of VPA in human plasma being 50*–*100 μg/mL, the sensitivity obtained from this analytical method was adequate for determining VPA in plasma (30 μg/mL). In addition, increased sensitivity might lead to increased interference or background noise. This method not only enhances the analysis of VPA in plasma but also indicates the potential for broader applications in drug quantification without the complexity of conventional sample preparation techniques.

## 5 Recommendations for future research

The developed HPLC-UV method for VPA detection in human plasma, utilizing MAD with PH HCl, while innovative, carries inherent limitations and disadvantages. Firstly, the specificity of MAD, although beneficial for VPA analysis, may not be directly applicable to other compounds, limiting its versatility. Furthermore, ensuring the complete solubility of reactants in acetonitrile poses a challenge, potentially affecting reaction efficiency and product yield. Lastly, the broader applicability of this method across different plasma matrices requires further validation to confirm its robustness and generalizability, indicating a need for comprehensive exploration in future studies. These limitations underscore the importance of ongoing research to refine and expand the method's utility in therapeutic drug monitoring. Future research may further explore the utilization of microwave assistance in pre-column derivatization, potentially transforming the sensitivity and efficiency of drug analysis in various laboratory settings.

## Funding statement

This study was financially supported by 10.13039/501100010034Walailak University, Thailand [grant numbers WU65233].

## Ethics statement

This research was approved by the Ethics Committee in Human Research at Walailak University (Ethical Approval Number: WUEC-23-194-01).

## Data availability statement

Data will be made available on request.

## CRediT authorship contribution statement

**Sirikanya Kaewpradit:** Writing – original draft, Visualization, Methodology, Funding acquisition, Formal analysis, Conceptualization. **Gorawit Yusakul:** Writing – review & editing, Visualization, Supervision, Resources, Methodology, Investigation, Formal analysis, Conceptualization. **Pornchai Rojsitthisak:** Writing – review & editing. **Chutima Jantarat:** Writing – review & editing, Visualization, Supervision, Resources, Project administration, Methodology, Investigation, Formal analysis, Conceptualization.

## Declaration of generative AI and AI-assisted technologies in the writing process

During the preparation of this work, the authors used ChatGPT 4.0 to improve language and readability. After using this tool, the authors reviewed and edited the content as needed and take full responsibility for the content published.

## Declaration of competing interest

The authors declare the following financial interests/personal relationships which may be considered as potential competing interests:

Sirikanya Kaewpradit reports financial support was provided by 10.13039/501100010034Walailak University. If there are other authors, they declare that they have no known competing financial interests or personal relationships that could have appeared to influence the work reported in this paper.
